# … Myself That I Remake

**DOI:** 10.3201/eid1502.000000

**Published:** 2009-02

**Authors:** Polyxeni Potter

**Affiliations:** Centers for Disease Control and Prevention, Atlanta, Georgia, USA

**Keywords:** Art–science connection, Lois Mailou Jones, African masks, American artists, painting, William Butler Yeats, facemasks, influenza, respiratory viruses, about the cover

**Figure:**
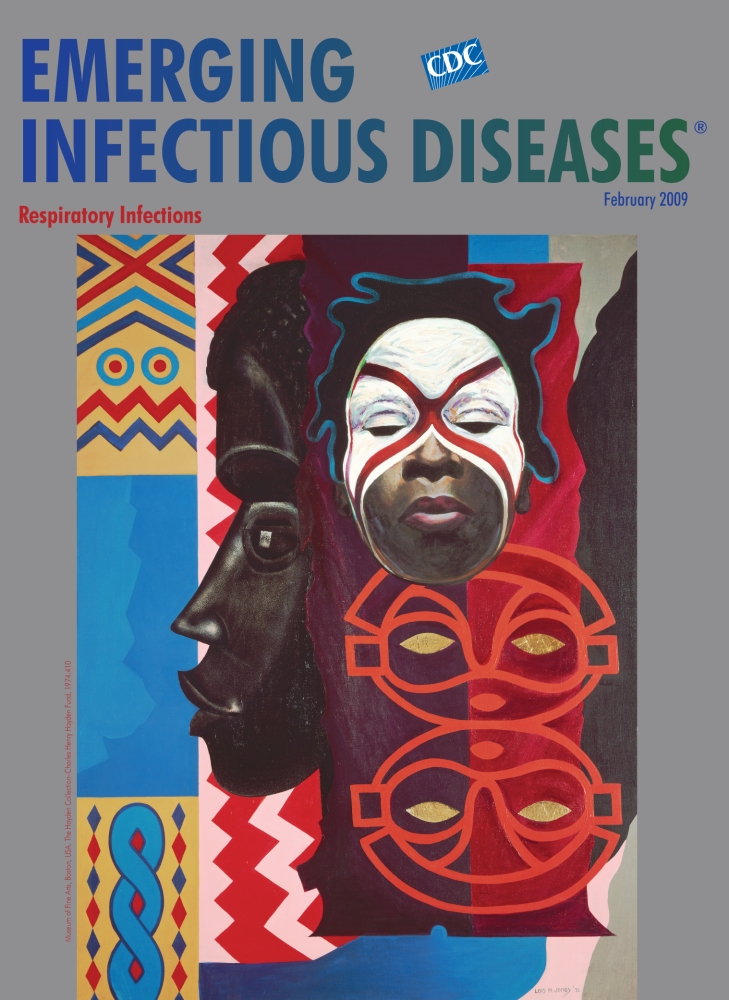
**Lois Mailou Jones (1905–1998). Ubi Girl from Tai Region (1972).** Acrylic on canvas (111.1 cm × 152.4 cm). Museum of Fine Arts, Boston, Massassachusetts, USA. The Hayden Collection―Charles Henry Hayden Fund, 1974.410

—W.B. Yeats

“I’m a lover of nature. I have to paint from within …. As I used to tell my students, anything I do must be of a caliber that will live after me. That is really my credo, even now,” said Lois Jones when she was in her 80s. During an artistic career that spanned more than 6 decades and continued until the end of her life, she sought excellence and a place in art history. When, in her early efforts, she realized that textile design entailed anonymity, she abandoned it. “As I wanted my name to go down in history, I realized that I would have to be a painter. And so it was that I turned immediately to painting.”

A Boston native, Jones received her early education locally, first at the High School of Practical Arts and in museum vocational drawing classes, later at the Boston Museum School of Fine Arts and the Designers Art School, where she studied textile design and created patterns for such textile manufacturers as F.A. Foster in Boston and Schumacher in New York City.

“Practically every summer of my childhood my mother took me and my brother to Martha’s Vineyard …. It was there that I first began to paint.” During these trips to the island Jones met two influential figures, sculptor Meta Warrick Fuller and composer Harry T. Burleigh. “Lois, you know, if you want to be successful in your career you’re going to have to go abroad,” they advised. Fuller had studied in Paris with Rodin, and Burleigh would regularly go to Switzerland to write his music. “Look what happened to Henry O. Tanner. He couldn’t make it in this country; he had to go abroad. The same thing happened to Hale Woodruff.”

Though Jones vowed to go abroad, Paris had to wait. After graduation from the Boston Museum School, she migrated south to North Carolina to teach at the Palmer Memorial Institute in Sedalia. Soon she was invited to Howard University in Washington, DC, where she founded the art department and taught design and watercolor painting for more than 40 years, making her mark and influencing hundreds of painters, among them Elizabeth Catlett, Alma Woodsey Thomas, and Malkia Roberts.

Jones got her big break in 1937, with a fellowship to study at the Académie Julien during a sabbatical from Howard. “I began to think of my dream coming true, of going to Paris, where I would be appreciated as an artist … and see the paintings of Lois Jones hanging beside those of artists from all over France and all over the world ….” In Paris, she gained confidence, met distinguished colleagues, and exhibited widely. “Impressionism was the way I worked … mostly with the palette knife.”

“When I returned from France … I missed all of that elegance which I had known in Paris ….” Under the new circumstances, “I owed very much to my white friend Céline who would take my paintings to the juries. They never knew that the artist was black. That was very much in my favor.”Jones returned to France often and went on other travels, notably to Africa and Haiti. She became comfortable in many styles and with portraits as well as landscapes. Ubi Girl from Tai Region, on this month’s cover, shows the influence of what was known as the Washington Color School, a group that favored precise motifs and bold flat fields of color. At a symposium in 1992, Jones described herself as the “only surviving painter of the Harlem Renaissance,” the movement of the 1920s and 1930s that celebrated African cultural identity and heritage.

“I bathed in the Euphrates when dawns were young/I built my hut near the Congo and it lulled me to sleep,” wrote Jones’ contemporary Langston Hughes, expressing his generation’s preoccupation with origins and identity. Jones delved into her own roots early in her career. “I can remember the students at Howard University saying that Professor [James A.] Porter, Professor [James Lesesne] Wells, and I didn’t appreciate African art. The students thought that they were the ones who were bringing African art into recognition. I told them that in 1937, before they were born, I had painted Les Fetiches ….”

“I found that the African masks gave me my best opportunity for studying the mask as a form, and my interest in the mask began very early in my career…. In Africa, I would go to the museums and make sketches and studies from the fetiches and the masks and use them in my creative paintings.” Jones was not alone in her fascination. In the 1900s, African sculpture became a strong force in the development of modern art, even though its original meaning and function were not known. In France, an underlying spiritual content was recognized. Stylized features were adapted and combined with postimpressionist elements from Cézanne and Gauguin to define the flat fragments of Henri Matisse, Pablo Picasso, and other pioneers of modernism.

Picasso described his bewildering first exposure to African masks in 1907. “When I went to the Trocadéro … a smell of mould and neglect caught me by the throat .... But I forced myself to stay, to examine these masks … that people had created with a sacred, magical purpose, to serve as intermediaries between them and the unknown …. And then I understood what painting really meant. It’s not an aesthetic process; it’s a form of magic that interposes itself between us and the hostile universe, a means of seizing power by imposing a form on our terrors as well as on our desires. The day I understood that, I realized that I had found my path.”

Masks have featured in many disciplines. William Butler Yeats (1865–1939) viewed them in the context of spiritual renewal. Like Picasso, he saw beyond aesthetics. He believed that artists are charged with perfecting themselves and the world and admonished “poet and sculptor” to perform this miracle. He was relentless in his own efforts. “The friends that have it I do wrong/When ever I remake a song,” he wrote, “Should know what issue is at stake:/It is myself that I remake.”

In many style transformations over her lengthy career, Jones did her part, remaking herself, revising, seeking new levels of perfection. The bold geometric designs of Ubi Girl from Tai Region, far removed from her early impressionist style, capture the underlying spirituality in the faces and masks she studied during her travels.

The Pythian expressions of the masks against the dark profile in the center of the painting challenge the viewer. What horror do they encase? What spirit do they conceal? Perhaps they shield against social injustice as experienced by Jones and so many others. Outside the artist’s realm and far from their origins, masks still serve as intermediaries between us and the unknown. In public health, they shield against nature’s horrors, respiratory viruses haunting our households. If worn consistently as designed, masks can protect against the flu and other infections. A new face we create for ourselves, they are still a “form of magic” pressed “between us and the hostile universe.”
